# N-Acetylcysteine Attenuates Aβ-Mediated Oxidative Stress, Blood–Brain Barrier Leakage, and Renal Dysfunction in 5xFAD Mice

**DOI:** 10.3390/ijms26094352

**Published:** 2025-05-03

**Authors:** Atcharaporn Ontawong, Geetika Nehra, Bryan J. Maloney, Chutima S. Vaddhanaphuti, Björn Bauer, Anika M. S. Hartz

**Affiliations:** 1 Sanders-Brown Center on Aging, College of Medicine, University of Kentucky, Lexington, KY 40536, USA; atcharaporn.ontawong@uky.edu (A.O.); geetika.nehra@nyulangone.org (G.N.); bryan.maloney@uky.edu (B.J.M.); bjoern.bauer@uky.edu (B.B.); 2 Division of Physiology, School of Medical Sciences, University of Phayao, Phayao 56000, Thailand; 3 Department of Physiology, Faculty of Medicine, Chiang Mai University, Chiang Mai 50200, Thailand; chutima.srimaroeng@cmu.ac.th; 4 Department of Pharmaceutical Sciences, College of Pharmacy, University of Kentucky, Lexington, KY 40536, USA; 5 Department of Pharmacology and Nutritional Sciences, University of Kentucky, Lexington, KY 40536, USA

**Keywords:** beta-amyloid (Aβ), blood–brain barrier (BBB), N-acetylcysteine, oxidative stress, 5xFAD, Alzheimer’s disease

## Abstract

Alzheimer’s disease (AD) is characterized by amyloid-beta (Aβ) pathology and is closely linked to oxidative stress, which contributes to blood–brain barrier leakage, renal dysfunction, and cognitive decline. We investigated the effects of N-acetyl cysteine (NAC), an FDA-approved antioxidant, on oxidative stress, brain Aβ levels, barrier leakage, renal function, and cognition in 5xFAD mice. Eight-week-old 5xFAD mice were fed a rodent diet supplemented with 600 mg/kg_Diet_ NAC for 4 weeks; wild-type (WT) mice and control 5xFAD mice were fed a regular rodent diet. We detected elevated brain and renal 4-hydroxynonenal(4-HNE) levels, reduced creatinine clearance, and increased plasma S100β levels in untreated 5xFAD mice compared to WT controls. Untreated 5xFAD mice also had higher capillary leakage, reduced P-gp activity, and impaired cognition compared to WT. NAC treatment of 5xFAD mice reduced brain Aβ40 levels, normalized 4-HNE levels to control levels, improved creatinine clearance, decreased capillary leakage, and lowered S100β plasma levels. NAC improved cognitive performance in 5xFAD mice, as shown by Y-maze. Our findings indicate that Aβ-induced oxidative stress contributes to barrier dysfunction, renal impairment, and cognitive deficits in 5xFAD mice. Notably, NAC treatment mitigates these effects, suggesting its potential as an adjunct therapy for AD and other Aβ-related pathologies by reducing oxidative stress.

## 1. Introduction

Alzheimer’s disease (AD) is one of the most common forms of dementia, characterized by a complex pathology that includes the accumulation of Aβ in extraneuronal plaques, neurofibrillary tangles, oxidative stress, synaptic loss, and neurodegeneration [1,2]. These pathological hallmarks contribute to cognitive decline and adversely affect patients’ quality of life [3,4]. Among these hallmarks, oxidative stress exacerbates AD pathology. The brain’s unique composition, characterized by high lipid content, elevated oxygen consumption, and a limited capacity for antioxidant defense, renders it vulnerable to oxidative damage [5,6]. This vulnerability is underscored by the fact that over 50 clinical trials have been initiated to explore the relationships between biomarkers of AD pathology, cognitive function, and oxidative stress [7]. Data from preclinical studies suggest that Aβ contributes to the generation of reactive oxygen species (ROS). ROS are implicated in a cascade that includes increased lipid peroxidation, oxidation of housekeeping proteins, altered enzymatic activity, and reduced overall brain metabolism [8,9].

Postmortem analysis of brain tissue from individuals with capillary cerebral amyloid (CAA) revealed the presence of Aβ plaques and ROS near disrupted tight junction proteins, underscoring the potential role of oxidative stress in the pathology of AD [10,11]. Epidemiological studies further support this association, indicating that a high intake of antioxidants correlated with a reduced risk of developing AD and related dementias [12]. Collectively, these findings highlight the intricate relationship between oxidative stress, Aβ pathology, barrier leakage, and cognitive decline in AD. However, the effects of antioxidants on AD are not well established. For example, vitamin C supplementation reduced amyloid plaque burden in the cortex and hippocampus in ι-gulono-γ-lactone oxidase knockout 5xFAD mice, ameliorating blood–brain barrier disruption and abnormal mitochondrial morphology [13]. A retrospective population study found no significant reduction in AD incidence among participants who took vitamin C alone. However, a combination of vitamin C and E was associated with reduced incidence [14]. A single-community prospective study reported that dietary vitamin E intake could reduce the risk of AD, whereas supplements alone did not show a benefit. In contrast, a later, larger meta-study indicated that both dietary and supplemental vitamin E may offer protective effects [15,16]. We selected N-acetyl cysteine (NAC) for our study based on its well-characterized pharmacological profile, ability to cross the blood–brain barrier, and dual antioxidant and neuroprotective properties. Its clinical safety has been established through decades of use in other indications, such as acetaminophen overdose and chronic respiratory diseases, making it a favorable candidate for translational research. NAC is a dietary supplement with a molecular weight of 163 Da, initially FDA-approved for medical use in patients with respiratory conditions, due to its mucus-clearing properties [17]. As a potent antioxidant and anti-inflammatory agent, NAC increases glutathione-S-transferase activity, scavenges free radicals, stabilizes protein structures through disulfide linkages, reduces proinflammatory cytokines, chelates heavy metals for excretion, reduces vascular permeability, and modulates neurotransmitters and neurotransmitter receptors in the brain [18,19,20].

Clinical trials have explored the effects of NAC on cognition, predominantly in conjunction with other dietary supplements for patients with mild cognitive impairment [21,22,23,24,25,26]. Notably, the impact of NAC on AD pathology, oxidative stress, and cognitive function remains largely unknown. Therefore, we aimed to evaluate the effects of dietary NAC exposure on brain Aβ levels, lipid peroxidation, barrier integrity, and renal function in 5xFAD mice.

The 5xFAD mouse model contains three autosomally-dominant, familial AD (FAD) mutations in the human amyloid precursor protein (APP) gene and two FAD mutations in the human presenilin 1 (PSEN1) gene [27]. These mice exhibit aggressive amyloid pathology and microvascular changes before the onset of spatial memory deficits and neuronal loss, making them a valuable tool for preclinical studies [27,28,29,30,31,32]. Microvascular abnormalities in this line include reduced cerebral blood flow, stalled brain capillaries, reduced capillary length, increased adhesion of neutrophils, and neurovascular inflammation [27,28]. The 5xFAD model represents early onset AD (EOAD), which covers 5% of all AD cases, but lacks the slow progression and neurofibrillary tangles found in sporadic AD. Like all AD animal models, the 5xFAD model does not fully capture the complexity of the human disease. Importantly for this study, however, 5xFAD mice are well suited for studying early (<3 months) Aβ-associated microvascular changes [33].

Emerging evidence suggests a critical role for renal function in the clearance of plasma Aβ [34]. For example, radiolabeled Aβ1–40 is detectable in both the kidney and urine after intravenous bolus injection [35]. A recent study found that the kidney clears Aβ peptides from the blood, implying that facilitating Aβ peptide clearance through the kidney might be a novel therapeutic strategy for AD [36]. Given the established link between oxidative stress and AD pathology, as well as the involvement of oxidative stress in chronic kidney disease and cognitive impairment, enhancing kidney function may represent a promising therapeutic strategy for AD [37,38]. Therefore, we hypothesized that NAC administration would attenuate oxidative stress, reduce lipid peroxidation, improve renal function, and mitigate blood–brain barrier leakage, ultimately improving cognitive performance in 5xFAD mice. Our study aims to elucidate the multifaceted effects of NAC on Aβ pathology and cognitive function, thereby contributing to understanding potential therapeutic avenues for AD.

## 2. Results

### 2.1. NAC Effect on Food Intake, Water Intake, Body Weight, and Plasma ALT Levels

**Figure 1:**
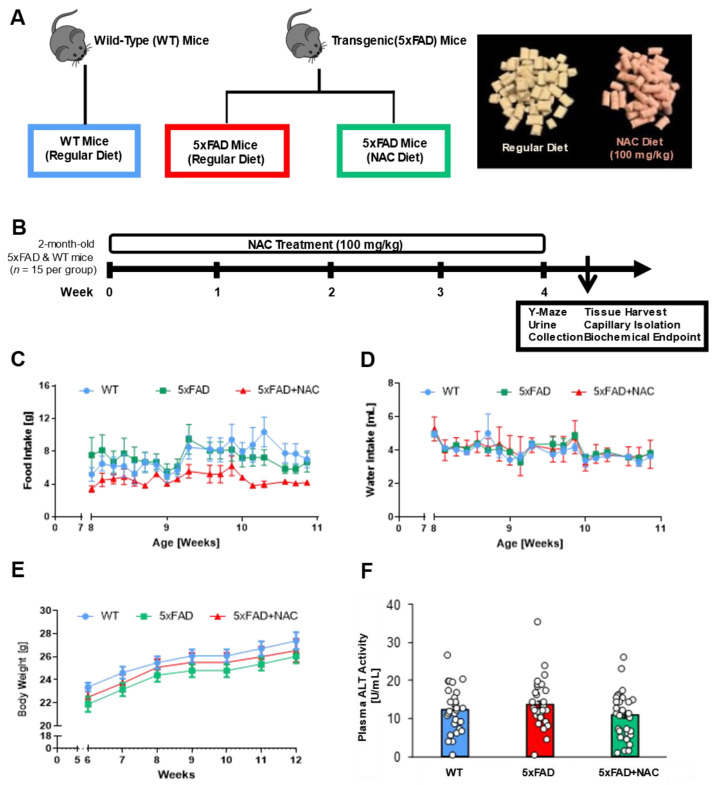
Experimental design, food and water intake, body weight, and plasma ALT activity. (**A**) Schematic diagram illustrating mouse genotypes and NAC treatment groups. Mice were divided into three groups (n = 15 mice per group): (1) wild-type (WT) mice, (2) 5xFAD mice on a regular diet, and (3) 5xFAD mice on an NAC-supplemented regular diet. The image shows color-coded food pellets used in the feeding study. (**B**) Male 5xFAD and WT mice were randomly assigned to their respective treatment groups at 8 weeks of age. After 4 weeks on the respective diet, mice underwent Y-Maze testing and urine collection, followed by tissue collection for biochemical endpoints. (**C**,**D**) Food intake (g/mouse) and water intake (mL/mouse) during the 4-week feeding study. (**E**) Body weight (g/mouse) during the feeding study. Blue circles, green squares, and red triangles show average data from untreated WT mice, untreated 5xFAD mice, and NAC-treated 5xFAD mice, respectively. (**F**) Plasma ALT activity (U/mL) for untreated WT mice (blue column), untreated 5xFAD mice (red column), and NAC-treated 5xFAD mice (green column). Circles represent technical replicates. Data are presented as mean (filled columns) ± standard error of the mean (SEM).

We examined the impact of the antioxidant NAC on blood–brain barrier integrity, kidney function, and cognition in 5xFAD mice. We exposed 8-week-old 5xFAD mice and WT littermates to a regular rodent diet or a diet containing 600 mg/kgDiet NAC (~100 mg/kg_Bodyweight_) for 4 weeks (Figure 1A,B). We had three treatment groups: (1) WT mice on a regular diet (*n* = 15), (2) 5xFAD mice on a regular diet (*n* = 15), and 5xFAD mice on a regular diet containing NAC (*n* = 15). We assessed each animal’s food intake, water intake, and body weight changes (Figure 1C–E). Overall, weekly food intake was the lowest for NAC-treated 5xFAD mice compared to 5xFAD and WT mice, but no significant differences were noted between any two groups (Figure 1C). Weekly water intake did not show significant differences across groups (Figure 1D). Weekly body weight measurements steadily increased for all treatment groups throughout the feeding study (Figure 1E).

To determine potential NAC-induced liver toxicity, we measured alanine aminotransferaxe (ALT) activity in plasma samples from 5xFAD and WT mice (Figure 1F). Mean plasma ALT activity was similar for untreated WT and untreated 5xFAD mice (WT: 12.3 ± 1.2 U/mL; 5xFAD: 13.6 ± 1.3 U/mL). Feeding 5xFAD mice with NAC diet did not alter ALT levels (10.9 ± 1.1 U/mL). The 4-week NAC treatment did not affect ALT activity, which indicates that NAC did not induce hepatotoxicity.

### 2.2. NAC Effect on Brain Lipid Peroxidation Levels

**Figure 2:**
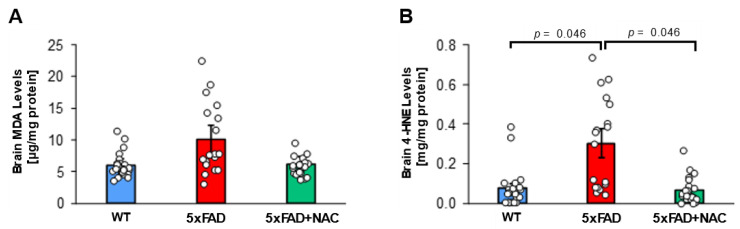
MDA and 4-HNE levels in 5xFAD and WT mice. (**A**) Brain MDA levels (μg/mg protein) for untreated WT mice (blue column; n = 15 mice), untreated 5xFAD mice (red column; n = 15 mice), and NAC-treated 5xFAD mice (green column; n = 15 mice). (**B**) Brain 4-HNE levels (mg/mg protein) were highest for untreated 5xFAD mice compared to untreated WT mice or NAC-treated 5xFAD mice (*p* = 0.046). Circles represent technical replicates. Data are presented as mean (filled columns) ± standard error of the mean (SEM).

Next, we assessed lipid peroxidation in 5xFAD and WT brain tissue samples. Mean brain malondialdehyde (MDA) levels for untreated 5xFAD mice (10.1 ± 0.9 μg/mg protein) were 1.7-fold higher than those of untreated WT mice (5.9 ± 0.5 μg/mg protein) and 1.6-fold higher than those of NAC-treated 5xFAD mice (6.2 ± 0.5 μg/mg protein; Figure 2A), but differences were not significant. Mean brain 4-HNE levels for untreated 5xFAD mice (0.3 ± 0.03 mg/mg protein) were 3-fold higher (*p* = 0.046) than mean brain 4-HNE levels for untreated WT mice (0.1 ± 0.01 mg/mg protein) and NAC-treated 5xFAD mice (0.1 ± 0.01 mg/mg protein; Figure 2B). Treating 5xFAD with NAC likely attenuated brain oxidative stress.

### 2.3. NAC Effect on Blood–Brain Barrier Function

**Figure 3:**
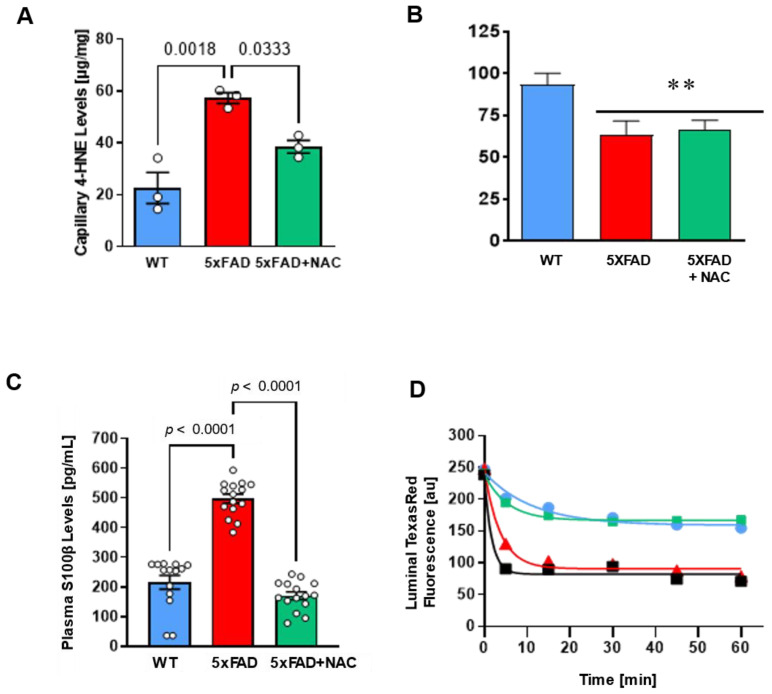
Effect of NAC on blood–brain barrier integrity in 5xFAD and WT mice. (**A**) Brain capillary 4-HNE levels (μg/mg protein) in untreated 5xFAD mice compared to those in untreated WT mice (*p* = 0.0018) or NAC-treated 5xFAD mice (*p* = 0.0333). Circles represent technical replicates. Data are presented as mean (filled columns) ± standard error of the mean (SEM). (**B**) Isolated capillaries were incubated with 2 μM NBD-CSA, a fluorescent P-glycoprotein-specific substrate, for 1 h alone or with PSC833. Specific luminal NBD-CSA fluorescence was taken as the difference between total luminal fluorescence and fluorescence in the presence of the P-gp inhibitor PSC833. Data are mean ± SEM for 10 capillaries from one preparation (pooled tissue from 15 mice per group), in arbitrary fluorescence units (scale 0–255). **, significantly lower than control, *p* < 0.01. (**C**) Plasma S100β levels (pg/mL) were 2.5-fold higher (*p* < 0.0001) for untreated 5xFAD mice (red column; n = 15 mice) than for untreated WT mice (blue column; n = 15 mice). NAC treatment (green column; n = 15 mice) significantly lowered S100β levels in 5xFAD mice (*p* < 0.0001). Circles represent biological replicates. (**D**) Texas Red leakage from capillary lumens was imaged over time using a confocal microscope for untreated WT mice (blue line), untreated 5xFAD mice (red line), and NAC-treated 5xFAD mice (green line). 100 mM mannitol served as a positive control for barrier opening (black line). Data are mean ± SEM for 7 brain capillaries per time point from one capillary isolation per group from 15 mice. Shown are arbitrary units (0–255). First-order efflux rates were calculated using non-linear regression.

We determined 4-HNE levels in isolated brain capillaries. 4-HNE levels in brain capillaries from untreated 5xFAD mice (57.26 ± 2.05 μg/mg protein) were 2.5-fold higher than levels from WT mice (22.60 ± 5.94 μg/mg protein) and NAC-treated 5xFAD mice (38.52 ± 2.45 μg/mg protein; Figure 3A). NAC reduced oxidative stress at the blood–brain barrier in 5xFAD mice. We also assessed (1) P-gp transport activity assay, (2) plasma S100β levels, and (3) capillary leakage assay using previously described assays [36,37,38,39,40]; Figure 3B–D). P-gp is a crucial transporter facilitating Aβ clearance across the blood–brain barrier [36]. Loss of P-gp in AD hinders Aβ removal and thus contributes to Aβ brain accumulation [41]. We, therefore, (Figure 3B–D). We assessed microvessels for P-gp transport activity. Isolated capillaries were incubated with NBD-CSA (P-gp substrate) for 1 h (steady-state) and were then imaged using confocal microscopy. Luminal NBD-CSA fluorescence in the capillary lumen was measured by quantitative image analysis using ImageJ. Luminal NBD-CSA fluorescence, an indirect measure for P-gp transport activity, was lower in capillaries from 5xFAD mice (63.40 ± 8.10 a.u.) compared to luminal fluorescence in WT capillaries (93.40 ± 6.70 a.u.). Luminal fluorescence in NAC-treated 5xFAD mice was comparable to that in untreated 5xFAD mice (66.20 ± 5.70 a.u.; Figure 3B), indicating that NAC did not affect P-gp transport activity.

S100β is a biomarker for barrier leakage that is predominantly expressed in astrocytes and readily detectable in peripheral blood when the blood–brain barrier is compromised [42]. We found that mean plasma S100β levels for untreated 5xFAD mice (497.1 ± 15.1 pg/mL) were 2.3-fold higher (*p* < 0.0001) compared to untreated WT mice (215.2 ± 22.9 pg/mL), indicating barrier leakage in these mice (Figure 3C). NAC-treated 5xFAD mice had similar plasma S100β levels (170.0 ± 12.7 pg/mL) to untreated WT mice, indicating that a 4-week NAC diet attenuated blood–brain barrier leakage in 5xFAD mice.

We followed up on these findings by assessing capillary leakage. In brief, isolated brain capillaries were loaded with fluorescent Texas Red (641 Da, 2 μmol/L) for 1 h. After washing with buffer, Texas red efflux from microvessel lumens was monitored by live cell imaging using confocal microscopy. Afterward, luminal Texas red fluorescence was quantified via image analysis software. We found that capillaries isolated from 5xFAD mice showed a 3.2-fold higher capillary leakage rate constant than those from WT mice (0.26 ± 0.03 min^−1^; Table 1). Capillaries isolated from NAC-treated 5xFAD mice had a capillary leakage rate constant (0.18 ± 0.03 min^−1^) comparable to WT mice (Table 1). Thus, NAC attenuates barrier leakage in 5xFAD mice but does not affect P-gp.

### 2.4. NAC Effect on Renal Function

**Figure 4:**
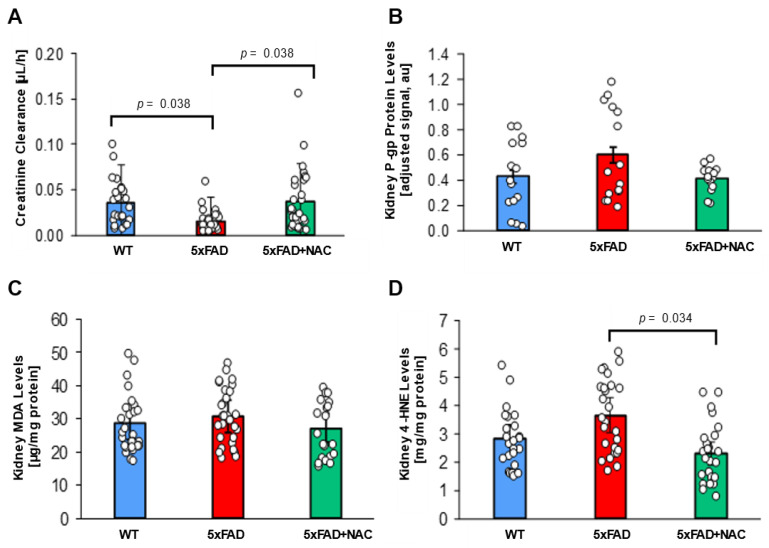
Creatinine clearance and renal P-gp levels in 5xFAD and WT mice. (**A**) Creatinine clearance (μL/h) was reduced for untreated 5xFAD mice (red column) compared to untreated WT mice (blue column; *p* = 0.038) and NAC-treated 5xFAD mice (green column; *p* = 0.038). (**B**) P-gp protein levels in crude kidney membrane fractions determined by Western blot followed by densitometric analysis (adjusted signal based on β-Actin, a.u.) were similar for untreated WT mice (blue column), untreated 5xFAD mice (red column), and NAC-treated 5xFAD mice (green column). Circles represent technical replicates. Data are presented as mean (filled columns) ± standard error of the mean (SEM). (**C**) Renal MDA levels (μg/mg protein) for untreated WT mice, untreated 5xFAD mice, and NAC-treated 5xFAD mice. (**D**) Elevated renal 4-HNE levels for untreated 5xFAD mice compared to the other two groups (*p* = 0.034). Circles represent technical replicates. Data are presented as mean (filled columns) ± standard error of the mean (SEM).

The kidney plays a significant role in Aβ elimination [31,43]. Thus, impaired renal function results in reduced Aβ clearance, which may contribute to its accumulation in the brain [44]. We assessed renal function in the mice by measuring plasma and urine creatinine levels by ELISA. Creatinine clearance was calculated using the following formula: [urine creatinine × urine volume)*/*(serum creatinine × urine collection time)]. Creatinine clearance in untreated 5xFAD mice (0.016 ± 0.002 μL/h) was 2.3-fold lower (*p* = 0.038) than that in untreated WT mice (0.036 ± 0.004 μL/h; Figure 4A). In contrast, creatinine clearance levels were similar for NAC-treated 5xFAD mice (0.037 ± 0.005 μL/h) and untreated WT mice, suggesting that NAC treatment rescued creatinine clearance in 5xFAD mice.

Using Western blotting, we also measured P-gp protein levels in 5xFAD and WT kidney cortices. We performed five runs; P-gp band intensities were normalized with corresponding β-actin band intensities. Adjusted P-gp signal was similar for untreated WT mice (8.0 ± 0.8 a.u.), untreated 5xFAD mice (11.1 ± 1.0 a.u.), and NAC-treated 5xFAD mice (7.7 ± 0.6 a.u.; Figure 4B). These findings show that NAC lowers elevated creatinine clearance in 5xFAD mice but does not impact renal P-gp expression.

We further determined MDA and 4-HNE levels in 5xFAD and WT mice kidney samples. Renal MDA levels for untreated 5xFAD mice (30.8 ± 0.03 μg/mg protein) were 1.1-fold higher than renal MDA levels for untreated WT mice (28.6 ± 2.4 μg/mg protein) and NAC-treated 5xFAD mice (27.2 ± 2.2 μg/mg protein; Figure 4C). Renal 4-HNE levels were also similar for untreated 5xFAD mice (3.6 ± 0.3 mg/mg protein) and untreated WT mice (2.8 ± 0.3 mg/mg protein; Figure 4D). These data indicate that lipid peroxidation products remained unchanged. NAC suppressed renal 4-HNE levels by 1.6-fold (*p* = 0.034; 2.3 ± 0.2 mg/mg protein) in 5xFAD mice. Together, these data indicate that 5xFAD mice exhibit elevated lipid peroxidation in brain and kidney tissue compared to WT mice, which can be restored to WT levels with an NAC-supplemented diet.

### 2.5. Multivariate Correlation Analysis of Protein Normalized Endpoints

**Figure 5:**
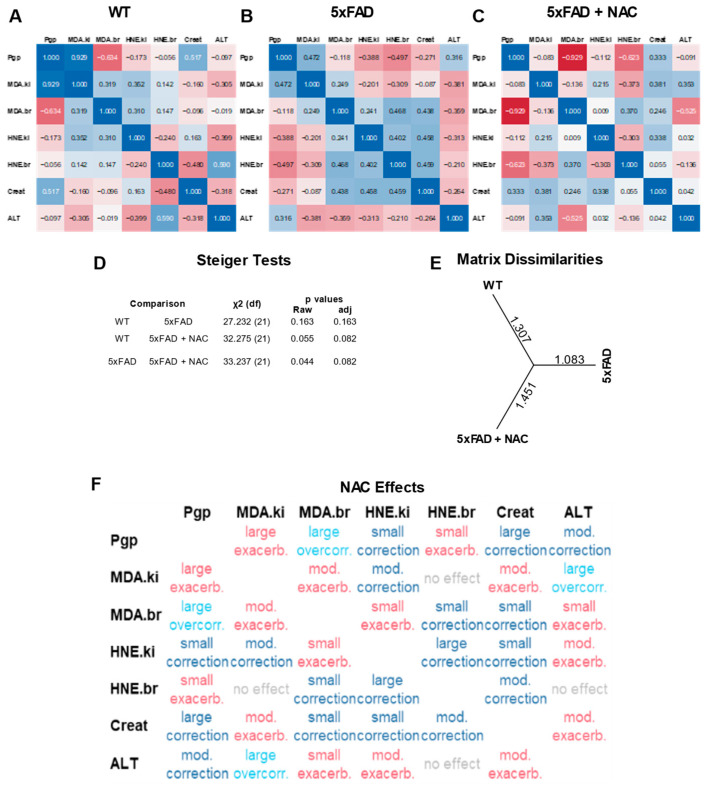
Multivariate correlation analysis of assay responses. (**A**) Correlation coefficients for renal P-gp levels (a.u.), renal MDA levels (μg/mg protein), brain MDA levels (μg/mg protein), renal 4-HNE levels (mg/mg protein), and brain 4-HNE levels (mg/mg protein) observed in WT mice. (**B**) Correlation coefficients for untreated 5xFAD mice. (**C**) Correlation coefficients for NAC-treated 5xFAD mice. (**D**) Steiger test results showing inter-group comparisons of correlation matrices. Overall, correlations were not significant in any group. (**E**) Matrix dissimilarities shown by Euclidean distances between groups. (**F**) NAC effects on 5xFAD correlation coefficients.

Figure 5A–C shows multivariate correlation analysis results of protein-normalized data from WT, 5xFAD, and NAC-treated 5xFAD mice. Each correlation matrix consists of seven outcomes measured in this study: (1) renal P-gp protein levels (a.u.), (2) renal MDA levels (μg/mg protein), (3) brain MDA levels (μg/mg protein), (4) renal 4-HNE levels (mg/mg protein), (5) brain 4-HNE levels (mg/mg protein), (6) creatinine clearance (μL/h), and (7) plasma ALT activity (U/mL). Overall, we observed no significant associations between outcomes within matrices. Steiger tests showed a distinct profile for NAC-treated 5xFAD mice compared to untreated WT and untreated 5xFAD mice, but the differences between matrices were non-significant (Figure 5D; *p* = 0.082). Assessment of matrix dissimilarities revealed similar Euclidean distances for NAC-treated 5xFAD mice (1.451) and WT mice (1.307) compared to untreated 5xFAD mice (1.083; Figure 5E), indicating that NAC shifted correlation coefficients in 5xFAD mice toward a WT profile. We further examined effect size and directions by computing grand differences of z-transformed correlation coefficients (Figure 5F). Overall, NAC-treated mice showed eight exacerbated outcomes (one large, four moderate, three small), eight corrected outcomes (four small, two moderate, two large), and two overcorrected outcomes. NAC effects had an overall score of 3.5 based on Table 2. In summary, multivariate correlation analysis showed a non-significant trend of restoring 5xFAD outcomes toward a profile similar to WT outcomes.

### 2.6. NAC Effect on Brain Aβ Levels

**Figure 6:**
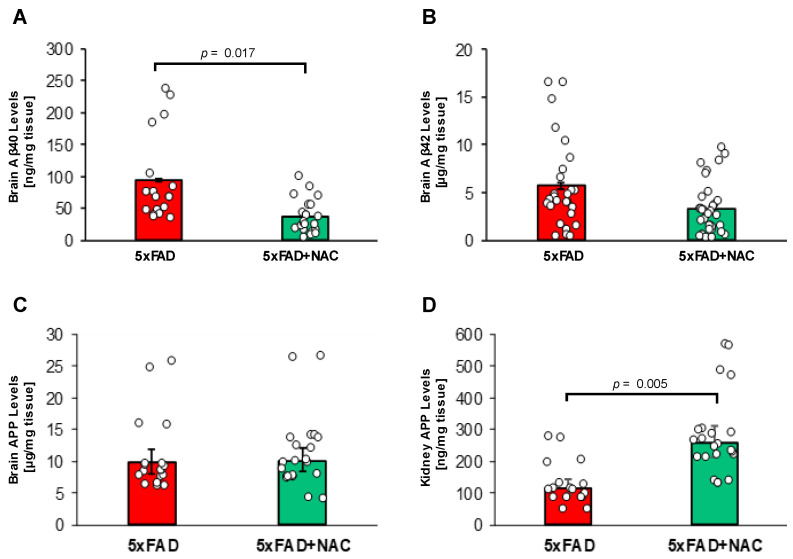
Brain Aβ levels and brain and kidney APP levels in 5xFAD and NAC-treated 5xFAD mice. (**A**) Mean brain Aβ40 levels (ng/mg tissue) were lower for NAC-treated 5xFAD mice (*p* = 0.017). (**B**) No changes in mean brain Aβ42 levels (μg/mg protein) were noted between groups. (**C**) NAC treatment did not associate with differences in brain APP levels (µg/mg tissue). (**D**) Levels of kidney APP were elevated (*p* = 0.005) in association with NAC treatment (ng/mg tissue). Circles represent technical replicates. Data are presented as mean (filled columns) ± standard error of the mean (SEM).

Figure 6A,B shows Aβ40 and Aβ42 levels in brain samples of untreated and NAC-treated 5xFAD mice measured by ELISA. Brain Aβ40 levels in NAC-treated 5xFAD mice (37.8 ± 3.9 ng/mg tissue) were 2.5-fold lower (*p* = 0.017) compared to those for untreated 5xFAD mice (94.6 ± 9.4 ng/mg tissue; Figure 6A). Mean brain Aβ42 levels in NAC-treated 5xFAD mice (5.7 ± 0.7 μg/mg tissue) were similar to levels found in untreated 5xFAD mice (3.3 ± 0.4 μg/mg tissue; Figure 6B). These data show that NAC treatment lowered brain Aβ40 levels in 5xFAD mice but did not affect brain Aβ42 levels. We found no significant differences in APP protein levels in the brain between untreated (9.8 ± 2.2 µg/mg tissue) and NAC-treated (10.0 ± 2.0 µg/mg tissue) mice. In contrast, NAC treatment was associated with a 2.2-fold increase in kidney APP (115.8 ± 26.2 vs. 258.1 ± 52.3 ng/mg tissue). However, APP kidney levels were 1000-fold lower compared to APP brain levels.

### 2.7. NAC Effect on Cognition

**Figure 7:**
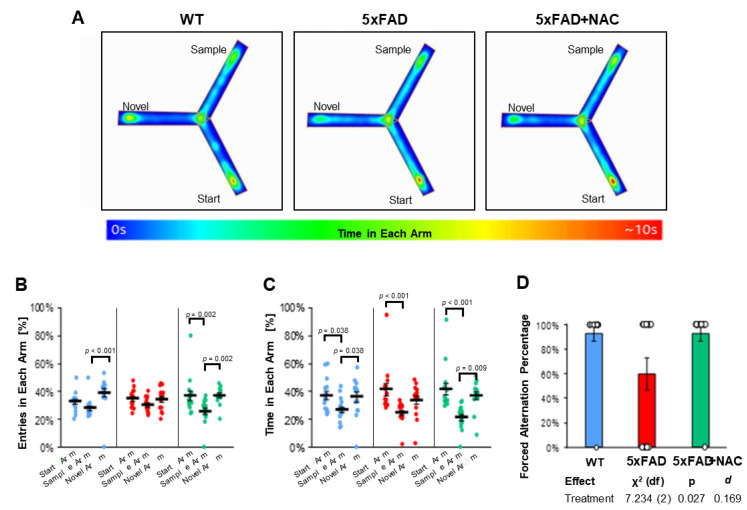
Y-maze test outcomes. (**A**) Heat maps showing the average time spent in each arm per treatment group, accompanied by a color-coded legend ranging from 0 to 10 s. (**B**) Entries in each arm (%) for untreated WT mice (blue dots), untreated 5xFAD mice (red dots), and NAC-treated 5xFAD mice (green dots). Entries varied across treatment groups (WT: *p* < 0.001 for sample vs. novel arm; 5xFAD: ns for all arms; 5xFAD + NAC: *p* = 0.002 for start vs. sample arm and novel vs. sample arm). (**C**) Time in each arm for all groups. Untreated mice spent the least time in the sample arm compared to the start or novel arm (*p* = 0.038 for both comparisons). Untreated 5xFAD mice spent more time in the start arm than the sample arm (*p* < 0.001). NAC-treated 5xFAD mice spent the least amount of time in the sample arm compared to the start arm (*p* < 0.001) or the novel arm (*p* = 0.009). (**D**) Forced alternation percentage for untreated WT mice (blue column), untreated 5xFAD mice (red column), and NAC-treated 5xFAD mice (green column). Data are presented as mean ± standard error of the mean (SEM).

Using the Y-maze test, we assessed 5xFAD and WT mice for their working memory and exploratory behavior [35]. Figure 7A shows representative heat maps of one mouse for each group during the 5 min retrieval trial, i.e., when all arms were accessible. Three parameters were analyzed from the retrieval trial data: (1) entries (%) in each arm (Figure 7B), (2) time (%) in each arm (Figure 7C), and (3) forced alternation percent (%, Figure 7D). Each of these parameters is discussed below.

Percent Entries in Each Arm: Mice in all groups showed 35–39% novel arm entries in the retrieval trial (Figure 7B). Untreated WT mice entered the novel arm more often (39.3% ± 3.0%; *p* < 0.001) than the start arm (32.7% ± 1.8%) or sample arm (28.0% ± 1.7%). In contrast, 5xFAD mice entered all arms equally (start arm: 35.0% ± 1.7%; sample arm: 30.5% ± 1.3%; novel arm: 34.5% ± 2.1%). NAC-treated 5xFAD mice entered start or novel arms (36.8% ± 3.5% and 37.3% ± 1.6%, respectively) equally and more often than the sample arm (25.9% ± 2.3%; *p* = 0.002). Post hoc analysis showed that arm entries were different across groups (omnibus *p* < 0.001) and that NAC significantly altered arm entry preferences toward the novel arm in 5xFAD mice (*p* = 0.021; Table 3).

Percent Time in Each Arm: Untreated WT mice spent 36–37% of their time in the start (37.2% ± 3.0%) and novel (36.0% ± 3.5%) arm and only 26.8% ± 2.2% of their time in the sample arm (*p* = 0.038; Figure 7C, Table 3). Likewise, untreated 5xFAD mice spent 41.5% ± 4.2% of their time in the start arm, 33.9% ± 2.9% in the novel arm, and 24.6% ± 1.9% in the sample arm. A similar pattern was observed for NAC-treated 5xFAD mice (41.6% ± 4.2% in the start arm; 37.1% ± 2.7% in the novel arm; 21.3% ± 2.2% in the sample arm). Post hoc analysis showed that time in arms was different across groups (omnibus *p* < 0.001), but NAC did not have a significant effect on time in arms for 5xFAD mice (Table 4).

Forced Alternations: Untreated 5xFAD mice showed 1.5-fold lower (60%) forced alternations than untreated WT mice (93%; Figure 7D). NAC treatment increased forced alternations in 5xFAD mice to 93%. Post hoc analysis of estimated marginal means showed that NAC significantly enhanced forced alternation indices (*p* = 0.027; Cohen’s d = 0.169; χ^2^ (df) = 7.234 (2); Figure 7D). Collectively, we found that NAC-treated 5xFAD mice showed increased novel arm preferences in forced alternations and arm entries in the Y-Maze test indicating that NAC treatment had a beneficial effect on cognition in 5xFAD mice.

## 3. Discussion

**Figure 8:**
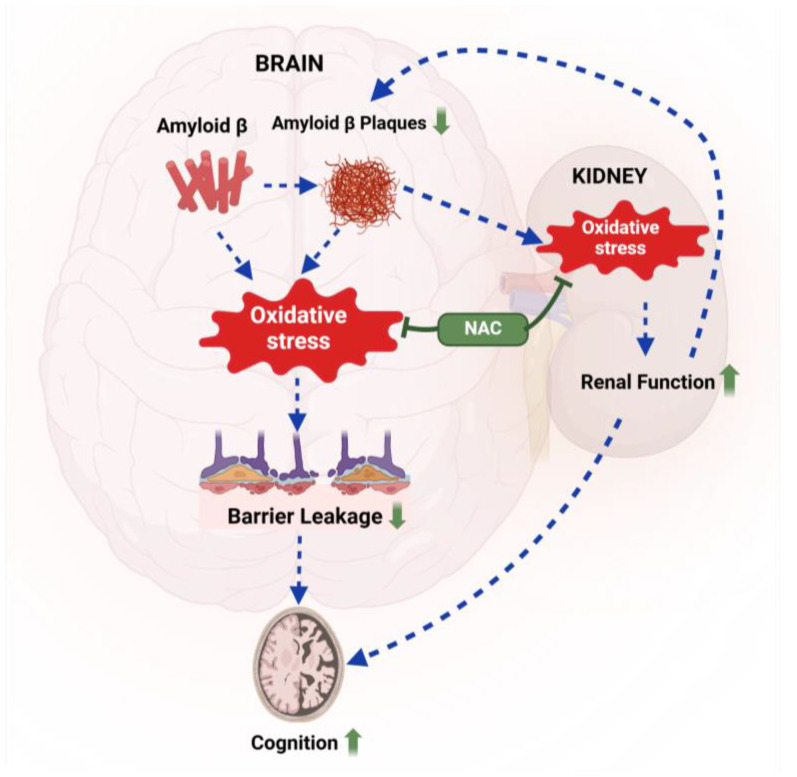
Proposed mechanism. Aβ-mediated oxidative stress triggers blood–brain barrier leakage, leading to cognitive impairment in 5xFAD mice. NAC treatment (green) reduces oxidative stress in the brain and the kidney, resulting in lower Aβ brain levels and improved cognitive performance in 5xFAD mice compared to untreated 5xFAD mice. Created with BioRender.com.

In this study, we assessed the effect of acute NAC treatment on brain Aβ levels, lipid peroxidation, blood–brain barrier integrity, renal function, and cognition in 5xFAD mice. At 12 weeks, 5xFAD mice showed increased lipid peroxidation, blood–brain barrier leakage, reduced creatinine clearance, and impaired exploratory behavior. Our findings provide proof-of-concept that short-term NAC treatment attenuates oxidative stress in 5xFAD mice, improves renal function, and reduces barrier leakage, which likely contributes to lower Aβ brain levels and improved cognition. Figure 8 provides a graphical summary of a potential mechanism based on our findings. Below, we discuss key aspects of this study in context with the existing literature.

*Brain Aβ Levels:* We found that a 4-week NAC treatment reduced brain Aβ40 levels in 5xFAD mice. Importantly, brain APP levels remained unchanged. NAC treatment is associated with elevated kidney APP in our work. This is the first report we are aware of that measured APP in the kidney after NAC treatment. Interpretation would require more in-depth study. Our findings are consistent with PET data that showed overlapping trajectories for oxidative stress and Aβ pathology PET tracers in 5xFAD mice between 2 and 12 months of age [41]. Four-month-old 5xFAD mice develop oxidized neuritis near Aβ plaques [42]. In vivo imaging showed that intracranial Aβ injection was sufficient to induce local oxidative stress in WT mice [45]. These reports support that Aβ pathology precedes oxidative stress in AD mouse models. Conversely, a detailed review stated that oxidative stress triggers cytoskeletal changes that impact APP processing, leading to increased Aβ pathology [46].

*Brain Lipid Peroxidation:* We found that NAC treatment reduced 4-HNE levels in the frontal cortices of 5xFAD mice to levels comparable to those in WT mice. Our findings are consistent with previous reports of elevated lipid peroxidation products in the brain tissue of 4–6-month-old 5xFAD mice. Notably, Western blot analysis [43] showed that 6-month-old 5xFAD mice exhibit significantly higher 4-HNE levels compared to their age-matched WT littermates. A 5-fold increase appeared in 4-HNE protein levels in brain tissue samples from 5xFAD compared to WT, along with a 10-fold higher 4-HNE immunoreactivity in frontal cortex neurons (area 2, medial prefrontal cortex) from 4-month-old 5xFAD mice, which was absent in age-matched WT mice [47].

Regarding MDA levels, we observed similar levels across groups with no statistically significant differences. This lack of significance may be attributed to the limited sensitivity and specificity of the TBARS assay [43,44]. Specifically, thiobarbituric acid reacts with various substances, such as sugars, amino acids, bilirubin, and albumin, which can differ between transgenic and non-transgenic strains, introducing artifacts in plate-based assays [43,48]. Additionally, hemolysis can vary in highly vascularized tissues (e.g., brain, kidney), further reducing the sensitivity of the TBARS assay [48]. Despite these limitations, prior studies using the TBARS assay have reported 1.5- to 3-fold higher MDA concentrations in brain tissue from 6-month-old 5xFAD mice compared to age-matched WT mice, indicating that MDA levels change with disease progression [49,50].

*Blood–Brain Barrier Leakage*: Our findings from isolated brain capillaries show that NAC treatment repairs barrier leakage in 5xFAD mice (Figure 3D). In vitro and in vivo data support the role of oxidative stress in compromising blood–brain barrier integrity through multiple mechanisms. First, in vitro data indicated that lipid peroxidation alters the phospholipid bilayer, altering membrane fluidity and permeability [51]. Second, oxidative stress activates MMP-9, which degrades tight junction and extracellular matrix proteins, thereby compromising the blood–brain barrier [52]. In vivo studies validated these findings, showing that antioxidants such as idebenone, crocin, and vitamin C upregulate certain blood–brain barrier proteins (e.g., low-density lipoprotein receptor-related protein 1 (LRP1), receptor for advanced glycation end products (RAGE) and P-gp), improve mitochondrial morphology, and increase tight junction length in 5xFAD mice [13,53,54]. However, the role of oxidative stress in driving barrier dysfunction is still not fully understood. Notably, we found that NAC had no effect on P-gp transport activity in isolated brain capillaries.

*Renal Function:* NAC reduced renal 4-HNE levels and increased creatinine clearance in 5xFAD mice (Figure 4). However, interpreting these results requires consideration of prior studies. C57BL/6 mice are resistant to renal injury and fibrosis [55,56]. An adenine-based diet generated a chronic kidney disease model in C57BL/6 mice, resulting in elevated BUN and serum creatinine levels [57]. Nine-month-old 5xFAD and WT mice had similar BUN and creatinine levels, but 5xFAD mice exhibited increased BUN and creatinine after 3 months on an adenine diet, whereas WT mice did not show such changes [58]. A separate study reported significant shrinkage, inflammation, and degeneration of the Bowman’s capsule and glomeruli in 24-month-old 5xFAD mice, which were absent in age-matched WT mice [59]. As there is limited consensus on renal dysfunction in younger 5xFAD mice, we urge caution in interpreting the extent of renal dysfunction in this model.

A positive correlation between Aβ levels and serum creatinine has been reported, suggesting that renal function is critical in maintaining Aβ homeostasis [60]. Clinical studies further support the association between renal dysfunction and the risk of dementia and Alzheimer’s disease (AD). High serum Aβ levels occurred in CKD patients, which correlated with renal dysfunction [36,37]. Furthermore, significantly elevated plasma Aβ and lipid peroxidation levels were found in CKD patients with cognitive impairment, alongside reduced levels of superoxide dismutase (SOD), catalase (CAT), glutathione peroxidase (GPx), and reduced glutathione (GSH) [61]. Cognitive decline in kidney disease also involves blood–brain barrier dysfunction. Recent data revealed that end-stage kidney disease patients exhibited higher blood–brain barrier permeability and lower Montreal Cognitive Assessment (MoCA) scores compared to healthy controls [62]. Together, these studies highlight that renal dysfunction impairs Aβ clearance, which contributes to blood–brain barrier dysfunction and AD pathology.

*Multivariate Profiling of NAC Biochemical Effects:* We did not stop at crude parallel presentation of various biochemical measures (P-gp, brain and kidney MDA, brain, and kidney HNE, creatinine, and ALT). We created profiles of their correlations within each treatment. While, due to sample size, individual correlations were not significant, Steiger’s testing of complete matrices revealed that the overall relationships among these outcomes differed by treatment. Scoring permitted observation that NAC-mediated correction vs. exacerbation of profiles in terms of restoration of similarity to WT from 5xFAD was a mixed bag, as it were. More studies with larger samples are probably needed. Multivariate analysis of outcomes is quite rare in the literature, and no comparative examples were found.

Our study represents an acute, proof-of-principle study focused on reducing oxidative stress via NAC treatment to achieve blood–brain barrier repair in AD. We acknowledge that AD is a long-term degenerative condition, and as such, our findings need to be validated in a long-term in vivo study to determine whether NAC confers any sustained benefits on Aβ pathology. Although our study showed reduced levels of brain Aβ, we did not examine corresponding changes in Aβ pathology, such as plaque burden. A longer-term investigation would be valuable to explore these outcomes. NAC is a sulfhydryl antioxidant, and while it serves as a useful starting point, other classes of antioxidants exist and may yield different outcomes. Comparative studies exploring the effects of different antioxidants would be highly informative. We also recognize that antioxidant therapies have not demonstrated consistent disease-modifying effects in clinical trials of AD.

This is also a pre-clinical study. While some studies have explored the use of NAC in individuals with pre-dementia, these studies have been small in scale and, in some cases, open-label [21,22,23,24,25,26]. We do not propose that NAC or similar agents should be adopted as a standalone treatment for AD. Rather, our work is focused on the repair of barrier dysfunction and how this is associated with apparent improvements in cognition. Future studies could benefit from comparing the effects of NAC in aged mice, modeling intervention during mild cognitive impairment versus AD. Evaluating NAC as an adjunct to currently approved therapies would provide valuable insights into its potential for combination treatment strategies. Translation to humans would, of course, require significant adjustment. For example, our work used a 600 mg/kg diet dose, which would translate to consumption of ~100 mg/kg/day in mice (3–4 g food eaten/day ÷ 20 g), which converts to ~8 mg/kg/day in humans (72 × 0.081 conversion factor) [63], which still would not consider the kinetics of once or twice-per-day dosing commonly used in human medication. In addition, the rate of NAC clearance in mice is over four times faster than in humans (34 min half-life vs. 2.27 h) [64,65].

In general, antioxidant-based interventions for AD have not yielded substantial clinical success [66,67]. Nevertheless, the field recognizes the potential of antioxidants. In fact, PubMed lists no fewer than 34 clinical trial reports on antioxidant use in AD within the past 5 years. A sampling of studies published in 2022–2025 highlights the wide range of compounds tested in the 5xFAD mouse model alone including tart cherry extract, Ω fatty acids, saponin, eugenitol, quercitrin, ergothioneine, hesperidin, oat seedling extract, Triphala, GHK peptide, Corinthian currants, Platycodon grandiflorum, ashwagandha, and “Asiatic acid” [68,69,70,71,72,73,74,75,76,77,78,79]. Each of these studies reported statistically significant results. However, none included direct comparisons to currently approved AD therapeutics, nor did any conduct head-to-head comparisons between antioxidants, which are limitations we acknowledge also in our study. While the diversity of tested antioxidants may be encouraging, it also underscores the absence of a consensus or gold standard. Although a post hoc meta-comparison might be tempting, it would lack reliability due to variations in study design, treatment protocols, and often limited sample sizes. Moreover, selecting a single comparator among such a broad array would ultimately be arbitrary. A robust, comparative analysis would require a comprehensive review specifically designed to address this complexity.

## 4. Materials and Methods

### 4.1. Chemicals

The fluorescent P-glycoprotein (P-gp) substrate, N-ε (4-Nitrobenzofurazan-7-yl)-d-Lys8]-cyclosporin A (NBD-CSA), was custom synthesized by R. Wenger (Basel, Switzerland) [80]. PSC833 was a kind gift from Novartis (Basel, Switzerland). NAC was purchased from ThermoFisher (160280250, ThermoFisher Scientific, Waltham, MA, USA). All other chemicals, reagents, buffers, and antibodies were obtained from commercial sources specified below.

### 4.2. Animals

Animal experiments were approved by the University of Kentucky’s Institutional Animal Care and Use Committee (Protocol #2014-1233; PI: AMS Hartz). Male 5xFAD mice (B6SJL-Tg (APPSwFlLon, PSEN1*M146L*L286V) 6799Vas/Mmjax; RRID:MMRRC_034840-JAX) and wild-type (WT) littermates were purchased from Jackson Laboratories (Bar Harbor, ME, USA). Mice were genotyped from ear punch samples by quantitative polymerase chain reaction (Transnetyx, Cordova, TN, USA). Mice were received at 8 weeks of age and group-housed in an AAALAC-accredited temperature-and-humidity-controlled housing room maintained at 25 ± 1 °C on a 14:10 h dark-light cycle with access to food and water ad libitum.

### 4.3. NAC Feeding Study

5xFAD mice and WT littermates were aged to 8 weeks and divided into three groups: (1) WT mice (n = 15) on a regular diet (Teklad^TM^ Global 18% Protein Rodent Diet No. 2918; Inotiv, West Lafayette, IN, USA), (2) 5xFAD mice (n = 15) on a regular diet, and (3) 5xFAD mice (n = 15) on a regular diet containing 600 mg/kg NAC. A regular diet containing 600 mg/kg NAC corresponds to an approximate daily intake of 100 mg/kg_Bodyweight_. This dosage was selected based on a prior study demonstrating that 100 mg/kg NAC effectively reverses memory impairment and reduces oxidative stress in the brain of aged mice [81]. 5xFAD and WT mice were exposed to either a regular or NAC diet for 4 weeks. Food, water intake, and health status were checked daily, and body weights were recorded weekly during the feeding study.

### 4.4. Y-Maze Test

5xFAD and WT mice were tested for spatial and working memory based on a modified Y-Maze test version as previously described [82]. In brief, forced and spontaneous alternations were tested in a Y-maze with three symmetrical arms (35 cm length × 5 cm width × 10 cm height; Part No. 60180; Stoelting Co., Wood Dale, IL, USA) where each arm was marked with black-and-white visual cues. Mice were handled and acclimated to test room conditions for three days before testing. Test room lighting conditions were set to 30 ± 5 lux to minimize anxiety behavior. The test consisted of a 5 min sample trial with the novel arm blocked and a 5 min retrieval trial with all arms unblocked. In the sample trial, mice were gently placed into the start arm and allowed to explore the start and sample arms during the trial. Mice were transferred to home cages for 30 min before the retrieval trial. In the retrieval trial, mice were placed in the start arm and were allowed to access all three arms. Animal performance in each trial was recorded using ANY-maze (version 499g Beta) an automated behavioral video tracking software (Stoelting, Wood Dale, IL, USA). Three parameters were measured from the recordings: (1) percent entries in each arm, i.e., entries in each arm divided by total arm entries; (2) percent time in each arm, i.e., time spent in each arm divided by total time in all arms; (3) forced alternation, i.e., percentage of mice with novel arm as first arm entry in retrieval trial.

### 4.5. Urine Collection

After Y-Maze testing, mice were housed in metabolic cages (Tecniplast, West Chester, PA, USA) for urine collection. Mice were acclimatized for 24 h before urine samples were collected. Urine flow rate was determined by dividing urine volume by collection time (24 h). Samples were centrifuged at 900× *g* and 4 °C for 15 min in a Fisherbrand^TM^ accuSpin^TM^ Micro 17R centrifuge (13-100-675; Fisher) and stored at −20 °C for creatinine assay.

### 4.6. Blood and Tissue Collection

Mice were euthanized by CO_2_ inhalation at the end of the NAC feeding study. Blood samples were collected from the lateral ventricle using a 20 GA PrecisionGlide^TM^ needle (305175, BD Biosciences, Franklin Lakes, NJ, USA). The abdominal cavity was opened to harvest kidney tissue. Blood samples were collected in 1 mL heparinized PSI microtainer tubes (365985; BD) and centrifuged at 5000× *g* for 5 min at 4 °C to collect plasma (supernatant). Plasma samples were stored at −20 C for S100β ELISA and ALT activity assay. Harvested kidney tissue was stored at −80 °C for TBARs assay, 4-HNE ELISA, and Western blotting. Frontal cortex brain tissue (~60 mg) was harvested, snap-frozen in liquid nitrogen, and stored at −80 °C for Aβ ELISA, TBARs assay, and 4-HNE ELISA. Remaining brain tissue was used for capillary isolation experiments.

### 4.7. Brain Capillary Isolation

Brain capillaries were isolated from whole mouse brain tissue samples as described previously [83,84]. Brain tissue was dissected, and cerebellum, olfactory bulbs, meninges, and white matter were discarded. Brains were pooled from the same treatment group, minced, and homogenized in ice-cold isolation buffer (Dulbecco’s phosphate buffered saline with 2.7 mM KCl, 1.46 mM KH_2_PO_4_, 136.9 mM NaCl, and 8.1 mM Na_2_HPO_4_ supplemented with 5 mM D-glucose and 1 mM sodium pyruvate, pH 7.4). Homogenization involved 100 strokes in a Potter-Elvehjem tissue grinder (clearance: 101–152 µm; 50 rpm) followed by 20 strokes in a hand-held Dounce homogenizer (clearance: 75–127 µm). The brain homogenate was mixed with 30% Ficoll^®^ (Millipore-Sigma, Burlington, MA, USA) and centrifuged at 5800× *g* for 15 min at 4 °C. Pellets were resuspended in 1% BSA and filtered through a 300 μm pluriStrainer^®^ (43-50300-03; PluriSelect, El Cajon, CA, USA) followed by three 30 μm pluriStrainer^®^ units (43-50030-03; PluriSelect). Strainers were inverted and rinsed with isolation buffer containing 1% BSA (A9647, Millipore-Sigma) to collect adhering capillaries for centrifugation (1500× *g*, 5 min, 4 °C). The final capillary pellet was resuspended in isolation buffer and used immediately for the capillary leakage and P-gp transport assays, as described below. The remaining capillaries were stored at −80 °C for 4-HNE ELISA.

### 4.8. P-gp Transport Assay

Isolated brain capillaries were assayed for P-gp transport activity as described previously [36,37,38]. In short, isolated capillaries were incubated in confocal imaging chambers with 2 μM NBD-CSA for 1 h at room temperature. A total of 10 to 15 capillaries were acquired per treatment group using a Leica TCS SP5 confocal microscope (0.63 × 1.2 NA water-immersion objective, 488 nm argon laser; Leica Instruments, Wetzlar, Germany). Images were analyzed for fluorescence intensities in the capillary lumen using ImageJ v1.45s (NIH, Bethesda, MD, USA). Specific luminal NBD-CSA fluorescence was determined as the difference between the total luminal fluorescence and fluorescence in the presence of the P-gp specific inhibitor PSC833 (5 μM final concentration).

### 4.9. Capillary Leakage Assay

Capillary leakage assay was performed as previously described [85,86,87,88]. In brief, freshly isolated brain capillaries were transferred to confocal chambers and loaded with 2 μM sulforhodamine 101 acid chloride (Texas Red; 641 Da; S3388; Millipore-Sigma) for 1 h at 25 °C. Texas Red-loaded capillaries were then washed with isolation buffer, and luminal Texas Red fluorescence was imaged over 60 min using a Leica TCS SP5 confocal microscope (0.63 × 1.2 NA water-immersion objective, 543 nm HeNe laser; Leica). For capillary leakage, 100 mM mannitol (M9546; Millipore-Sigma) was a positive control. Images of 10–15 capillaries per chamber were acquired, and luminal fluorescence was quantified using ImageJ v1.48s. First-order efflux rate constants were calculated by non-linear regression (Prism v7; GraphPad Software, San Diego, CA, USA).

### 4.10. Crude Kidney Membrane Isolation

Kidney cortices were thawed and resuspended in 0.5 mL CelLytic^TM^ lysis buffer (C2978; Millipore-Sigma) supplemented with 5× cOmplete^TM^ protease inhibitor cocktail (11697498001; Millipore-Sigma). Resuspended samples were transferred to 3.5 mL polycarbonate tubes (349622; Beckman Coulter, Indianapolis, IN, USA) and homogenized at 30,000 rpm for 200 s using a Polytron 2500E homogenizer (Kinematica, Bohemia, NY, USA). Capillary pellets were homogenized using a 5 mm probe (PT-DA 05/2EC-F078; Kinematica). Kidney samples were homogenized using a 3 mm probe (Kinematica). Homogenates were centrifuged at 30,000 rpm for 30 min at 4 °C in a TLA 100.2 fixed-angle rotor in a benchtop ultracentrifuge (359732; Beckman Coulter). Supernatants were transferred to clean polycarbonate tubes and centrifuged at 95,000 rpm for 2 h at 4 °C. After centrifugation, pellets were resuspended in LPC buffer (HyClone PBS, SH30256.01, Cytiva, Marlborough, MA, USA; CelLytic^TM^ lysis buffer, C2978; Millipore-Sigma; 1:1 volume ratio; supplemented with cOmplete^TM^ protease inhibitor cocktail, 11697498001, Millipore-Sigma, 5× final concentration). Resuspension volumes were 40 μL per sample for crude kidney membrane fraction. Resuspended pellets were frozen at −20 °C for Western blotting.

### 4.11. Western Blotting

Protein expression levels for crude kidney membrane fraction samples were assessed by Western blotting using the Invitrogen NuPAGE^®^ Novex^®^ electrophoresis and blotting system (Invitrogen, Carlsbad, CA, USA). All samples were first analyzed for protein concentrations using the Bradford assay [89]. For crude kidney membrane samples, protein concentrations were adjusted to 0.1 μg/μL (1.8 μg total protein per well). Samples were mixed with 4× NuPAGE^TM^ LDS Sample Buffer (Invitrogen) and electrophoresed on a 4–12% Bis-Tris gradient gel (Invitrogen) using the X-Cell SureLock^TM^ Mini-Cell (Invitrogen). Electrophoresis was done at a constant voltage of 200 V until complete separation of the molecular weight ladder (Rainbow^TM^, GE Healthcare Biosciences, Piscataway, NJ, USA). After electrophoresis, proteins were transferred to a polyvinylidene difluoride membrane (Invitroge) at 30 V for 2 h. Membranes were blocked with protein-free T20 blocking buffer (Pierce, Waltham, MA, USA) and incubated overnight at 4 °C with primary antibodies (P-gp: 0.3 μg/mL final concentration, 1:1000 dilution, ab170904, Abcam, Cambridge, MA, USA; β-actin: 0.3 μg/mL final concentration, 1:1000 dilution, ab8226, Abcam). Incubated membranes were washed and incubated with horseradish peroxidase-conjugated ImmunoPure^TM^ goat-anti-rabbit IgG (111-035-003; 1:10,000 dilution; 80 ng/mL final concentration; Pierce) and horseradish peroxidase-conjugated ImmunoPure^TM^ goat-anti-mouse IgG (115-035-003; 1:10,000 dilution; 80 ng/mL; Pierce) for 1 h at room temperature. Protein bands were detected with Pierce SuperSignal^TM^ West Pico PLUS Chemiluminescent Substrate (A32955; Pierce) on a ChemiDoc^TM^ XRS imager (Bio-Rad Laboratories, Hercules, CA, USA). Band intensities were measured with ImageLab software (v.4.6.9; Bio-Rad Laboratories, Hercules, CA, USA).

### 4.12. Aβ Extraction

Brain tissue samples from individual 5xFAD mice were weighed, recorded, and homogenized in 0.3 mL extraction buffer A (5 M Guanidine-HCl, G3272, Millipore-Sigma; 50 mM Tris HCl, T0819, Millipore-Sigma; pH 8) to extract total Aβ protein. Homogenates were diluted at a 1:10 ratio in extraction buffer B (DBPS, D5652, Millipore-Sigma; 5% BSA, A9647, Millipore-Sigma; 1× Protease Inhibitor Cocktail Set 1; Calbiochem, 539131, EMD Millipore, Billerica, MA, USA; 0.3% Surfact-Amps^TM^ 20, 28320, Fisher) and centrifuged at 16,000× *g* for 20 min at 4 °C. Supernatants containing Aβ were stored at −20 °C.

### 4.13. Aβ40 and Aβ42 ELISA

Human Aβ40 and Aβ42 levels were measured in brain samples using commercial enzyme-linked immunosorbent assay (ELISA) kits according to the manufacturer’s protocol (Aβ40: KHB3481 and Aβ42: KHB3441; Invitrogen). Brain homogenate samples were plated at 1:100 and 1:1000 dilutions in sample diluent buffer for Aβ40 and Aβ42 ELISA, respectively. Absorbance values were measured at 450 nm using the Synergy H1 microplate reader (Serial 15011310, BioTek^®^ Instruments, Winooski, VT, USA). Standard curves were generated using Gen5 software with non-linear 4-parameter-logistic regression curve fitting to determine Aβ40 and Aβ42 levels.

### 4.14. S100β ELISA

Plasma S100β levels were determined using a commercially available ELISA kit (EZHS100B-33K, Invitrogen) based on the manufacturer’s protocol. Plasma samples were centrifuged at 2500× *g* for 10 min at 4 °C and protease inhibitor (Protease Inhibitor Cocktail Set I (539131, Millipore-Sigma; 1:50 dilution, i.e., 2 μL in 100 μL) was added to the supernatant. Undiluted plasma samples were plated, and absorbance values were measured at 450 and 590 nm using a Synergy H1 plate reader (Serial 15011310, BioTek). Absorbance values at 450 nm were corrected with reference values of corresponding wells at 590 nm. Standard curves were computed using Gen5 software with non-linear 4-parameter-logistic regression curve fitting to determine S100β levels.

### 4.15. Brain Homogenization for TBARs Assay and 4-HNE and APP ELISAs

Brain tissue (40–45 mg) and kidney cortex (40–45 mg) were minced and homogenized on ice in 0.4 mL RIPA Buffer (250–500 μL; #R0728, Millipore-Sigma) containing 1× complete protease inhibitor cocktail (10659100, Roche Applied Science, Indianapolis, IN, USA; 1 μL in 100 μL). Samples were centrifuged at 1600× *g* for 10 min at 4 °C. Supernatants were collected and stored at −80 °C for TBARs assay, 4-HNE ELISA, and APP ELISA.

### 4.16. TBARS Assay

Brain and kidney tissue samples were assayed for lipid peroxidation using a commercial TBARS assay kit (10009055, Cayman Chemical, Ann Arbor, MI, USA) per the manufacturer’s protocol. Thawed samples were mixed with SDS solution and color reagent, boiled for one hour, and placed on ice for 10 min to stop the reaction. Samples were centrifuged at 1600× *g* for 10 min at 4 °C and plated on a 96-well plate for final readings. Absorbance values were measured at 540 nm using the Synergy H1 plate reader. MDA concentrations in the supernatant were determined using Gen5 software with non-linear 4-parameter-logistic regression curve fitting. MDA levels were normalized for total protein levels as determined by Bradford Assay.

### 4.17. APP ELISA

Levels of brain and kidney amyloid precursor protein (APP) were determined via a commercial APP ELISA kit (ab216944, Abcam). Thawed samples were diluted 1:1000 for brain samples and 1:5 for kidney samples in Cell Extraction Buffer PTR. All samples and standards were plated in a 96-well plate according to the manufacturer’s protocol and measured at 450 nm with a Synergy H1 plate reader. Brain and kidney APP levels were evaluated using Gen5 software with non-linear 4-parameter-logistic regression curve fitting.

### 4.18. 4-HNE ELISA

The lipid peroxidation product, 4-hydroxy-2-nonenal (4-HNE), was assessed in brain capillary, brain, and kidney tissue samples using a commercial 4-HNE ELISA kit (ab238538, Abcam) according to the manufacturer’s protocol. Thawed samples were diluted to a 1:10 ratio for brain and kidney samples in assay diluent buffer and plated in a 96-well plate. Absorbance values were measured at 450 nm using a Synergy H1 plate reader. 4-HNE levels in the supernatant were determined using Gen5 software with non-linear 4-parameter-logistic regression curve fitting. Total protein levels were determined by Bradford assay for normalizing 4-HNE levels.

### 4.19. ALT Assay

Alanine transaminase (ALT) activity was measured in plasma samples using the ALT activity assay kit (700260, Cayman Chemical, Ann Arbor, MI, USA) according to the manufacturer’s instructions. Undiluted plasma samples were incubated with 150 μL ALT substrate solution and 20 μL ALT cofactor solution in a 96-well plate at 37 °C for 15 min. After 15 min, 20 μL ALT initiator solution was added to each well to initiate the reaction. Absorbance values were measured at 340 nm once every minute over a 10 min duration using a Synergy H1 plate reader. The amount of generated pyruvate, a measure of ALT activity, was calculated from the standard curve using the following equation: [{(A_340nm,0min_ − A_340nm,5min_)/5 min} × 0.21 mL]/(4.11 mM^−1^ × 0.02 mL). Here, A_340nm_ is absorbance at 340 nm, 4.11 mM^−1^ is adjusted NADH extinction coefficient, 0.21 mL refers to total assay volume (0.02 mL sample + 0.15 mL ALT substrate solution + 0.02 mL ALT cofactor solution + 0.02 mL ALT initiator solution), and 0.02 mL indicates sample volume. ALT activity is reported as μmol/min/mL or unit/mL (U/mL), where one unit (U) of ALT is defined as the amount of enzyme that generates 1 μmol pyruvate per minute at 37 °C.

### 4.20. Creatinine Clearance

Per the manufacturer’s instructions, urine samples were tested for creatinine levels using the Creatinine (urinary) Colorimetric Assay kit (500701, Cayman Chemical, Ann Arbor, MI, USA). Samples were plated undiluted to ensure creatinine concentration values fall within the standard curve range (0–15 mg/dL). Alkaline picrate solution was added to initiate the formation of the red-colored creatinine–picrate complex. Plasma samples were tested for creatinine levels using the Creatinine (serum) Colorimetric Assay kit (700460, Cayman Chemical, Ann Arbor, MI, USA) according to the manufacturer’s instructions. Absorbance values of the creatinine–picrate complex in wells were measured at 490 nm using a Synergy H1 microplate reader (Serial #15011310, BioTek). A standard curve was generated using BioTek^®^ Gen5 Software v2.07 (Agilent, Santa Clara, CA, USA) using 4-PL curve fitting for determining creatinine concentrations in urine samples. Creatinine clearance was calculated using the following formula: [urine creatinine × urine volume)/(plasma creatinine × 1440 min)].

### 4.21. General Statistical Analysis

The following responses were analyzed for the effect of NAC treatment: P-gp protein expression, brain MDA levels, brain 4-HNE levels, creatinine clearance, brain Aβ40 levels, plasma Aβ40 levels, brain Aβ42 levels, plasma Aβ42 levels, and liver ALT activity. Responses were analyzed by mixed-level generalized linear models to account for technical replicates, followed by analyses of variation (ANOVA). *p*-values were adjusted by Benjamini-Hochberg correction for false discovery rate from 11 simultaneous comparisons [90]. Pairwise differences were assessed from estimated marginal means with Benjamini-Hochberg adjustments for three simultaneous comparisons [91]. Models were assessed as appropriate for meeting standard statistical assumptions, such as independence and identical distribution, e.g., normality plots of residuals [92].

### 4.22. Multivariate Correlation Analysis

Within each treatment group, correlation coefficients were calculated for all combinations of responses to generate correlation matrices. Aβ-related responses were excluded from this analysis due to the absence of Aβ measurements from WT mice. Correlation matrices were compared for significant differences by Steiger tests [93]. *p*-values were adjusted by Benjamini-Hochberg correction for simultaneous pairwise comparisons. Matrix dissimilarities were estimated by Euclidean distances of z-transformed correlation coefficients and plotted as a neighbor-joining unrooted tree. Directions of NAC effects were determined as (1) correction (i.e., NAC reduced 5xFAD effects), (2) exacerbation (i.e., NAC increased 5xFAD effects), and (3) overcorrection (i.e., NAC reduced 5xFAD effects, and values from NAC-fed mice exceed values from untreated 5xFAD mice). The extent of NAC effects was classified based on the grand difference of z-transformed coefficients, i.e., [(Coefficient_5xFAD_ − Coefficient_WT_) − (Coefficient_5xFAD+NAC_ − Coefficient_WT_)]. Grand differences were back-transformed, categorized, and scored based on Table 1 to determine the category of effect extent. Grand differences between 0.1 and 0.3, 0.3 and 0.5, and greater than 0.5 were classified as small, moderate, and large effects, respectively.

## 5. Conclusions

In conclusion, our study highlights the critical role of Aβ-induced oxidative stress in driving barrier dysfunction, renal impairment, and cognitive deficits in AD. Our findings provide evidence of the impact of Aβ pathology beyond the brain, emphasizing the need for a broader perspective in AD research. Importantly, NAC treatment was effective in attenuating these detrimental effects, suggesting its potential as an adjunct therapy for AD and other Aβ-related disorders. To provide context for our work, it is important to note that AD research, when it addresses the blood–brain barrier, typically focuses on “overcoming” it, rather than supporting or repairing it. The blood–brain barrier is often portrayed as a passive obstacle to treatment, rather than a vital component of healthy brain function. While we conducted standard assays common to AD animal model research, our study extended beyond them. We demonstrated that the outcomes of these assays correlated with blood–brain barrier repair, highlighting its role in disease progression and treatment response. Despite its importance, therapeutic strategies that protect or repair the blood–brain barrier do not exist. Our findings support the potential of targeting oxidative stress to mitigate Aβ-related pathology, including cognitive decline, Aβ accumulation, and blood–brain barrier dysfunction. However, further research is needed to identify the underlying mechanisms and the most promising therapeutic approaches. Future studies should compare the efficacy of several antioxidants and evaluate their use in combination with current standard AD treatments.

## Figures and Tables

**Table 1:** Texas red leakage rates for 5xFAD and WT capillaries.

Group	k (min^−1^)
WT	0.08 ± 0.01
5xFAD	0.26 ± 0.03
5xFAD + NAC	0.18 ± 0.03
100 mM Mannitol	0.58 ± 0.12

**Table 2:** Scoring of correlation categories.

Category	Score
Large Exacerbation	−3.0
Moderate Exacerbation	−2.0
Small Exacerbation	−1.0
No Effect	0.0
Small Correction	1.0
Moderate Correction	2.0
Large Correction	3.0
Small Overcorrection	0.5
Moderate Overcorrection	1.0
Large Overcorrection	1.5

**Table 3:** ANOVA for arm entries (%) in Y-maze test.

Effect	χ^2^ (df)	*p*	*R* ^2^
Treatment	0.236 (2)	0.888	0.030
**Arm**	**27.488 (2)**	**<0.001**	**0.195**
**Treatment × Arm**	**11.552 (4)**	**0.021**	**0.031**
**Omnibus**	**40.212 (8)**	**<0.001**	**0.195**

Chi-squared (χ^2^) values, degrees of freedom (df), *p*-values (adjusted by Benjamini-Hochberg false discovery correction), and *R*^2^ values. Relevant effects with *p* < 0.05 are discussed in the results section and shown in bold text here.

**Table 4:** ANOVA for time in each arm (%) in Y-maze test.

Effect	χ^2^ (df)	*p*	*R* ^2^
Treatment	0.252 (2)	0.881	0.015
**Arm**	**7.001 (2)**	**0.030**	**0.259**
Treatment × Arm	4.464 (4)	0.347	0.014
**Omnibus**	**38.777 (8)**	**<0.001**	**0.259**

Chi-squared (χ^2^) values, degrees of freedom (df), *p*-values (adjusted by Benjamini-Hochberg false discovery correction), and *R*^2^ values. Relevant effects with *p* < 0.05 are discussed in the results section and shown in bold text here.

## Data Availability

The raw and analyzed datasets generated for this study are available from the corresponding author upon reasonable request.

## Abbreviations

4-HNE	4-Hydroxynonenal
AD	Alzheimer’s disease
ALT	alanine transaminase
APP	amyloid precursor protein
Aβ	amyloid beta
ELISA	enzyme-linked immunosorbent assay
MDA	malonaldehyde
NAC	N-acetylcysteine
NBD-CSA	N-ε (4-nitrobenzofurazan-7-yl)-D-Lys8]-cyclosporin A
P-gp	P-glycoprotein
PSEN1	presenilin 1
TBARS	thiobarbituric acid reactive substances
WT	wild-type
